# Analysis of genomic signatures in prokaryotes using multinomial regression and hierarchical clustering

**DOI:** 10.1186/1471-2164-10-487

**Published:** 2009-10-21

**Authors:** Jon Bohlin, Eystein Skjerve, David W Ussery

**Affiliations:** 1 Norwegian School of Veterinary Science, P.O. Box 8146 Dep., N-0033 Oslo, Norway; 2 Center for Biological Sequence Analysis, Technical University of Denmark, DK-2800 Lyngby, Denmark

## Abstract

**Background:**

Recently there has been an explosion in the availability of bacterial genomic sequences, making possible now an analysis of genomic signatures across more than 800 hundred different bacterial chromosomes, from a wide variety of environments.

Using genomic signatures, we pair-wise compared 867 different genomic DNA sequences, taken from chromosomes and plasmids more than 100,000 base-pairs in length. Hierarchical clustering was performed on the outcome of the comparisons before a multinomial regression model was fitted. The regression model included the cluster groups as the response variable with AT content, phyla, growth temperature, selective pressure, habitat, sequence size, oxygen requirement and pathogenicity as predictors.

**Results:**

Many significant factors were associated with the genomic signature, most notably AT content. Phyla was also an important factor, although considerably less so than AT content. Small improvements to the regression model, although significant, were also obtained by factors such as sequence size, habitat, growth temperature, selective pressure measured as oligonucleotide usage variance, and oxygen requirement.

**Conclusion:**

The statistics obtained using hierarchical clustering and multinomial regression analysis indicate that the genomic signature is shaped by many factors, and this may explain the varying ability to classify prokaryotic organisms below genus level.

## Background

The lowering sequencing costs are resulting in an exponentially increasing amount of available genetic data [1]. The increase in genomic data is rapidly approaching the limit of what is possible to handle using today's computers. To overcome this challenge, the focus is shifting towards the development of methods capable of analyzing genomic data fast and efficiently. The advancement in sequencing technology is also responsible for the rapidly increasing field of metagenomics. Metagenomics is the study of genetic material taken from microorganisms living in different environments. The field of metagenomics gives researches access to the genetic contents of all organisms in an environment, including a wide variety of previously uncultivable organisms [2]. Metagenomic samples may therefore consist of genomic DNA sequences with no homology matches or known taxonomic marker genes. Methods that can classify unknown DNA sequences are therefore of great interest to metagenomic research [3].

In the present work we examine the "genomic signature" of an organism that can be found in an arbitrary fraction of genomic DNA using dinucleotide relative abundance patterns [4]. By dividing genomic dinucleotide frequencies with the corresponding mononucleotide content, Karlin and co-workers found a strong phylogenetic signal in the organisms tested. This signal was therefore referred to as a genomic signature [4]. An alternative view of this approach is that genomic AT content bias is removed from DNA word frequencies. This gives an odds-ratio of observed divided by approximated oligonucleotide frequencies. Comparing prokaryotes using genomic signatures can be considered as a measure of how DNA words are over- or underrepresented within genomes from what is expected from genomic AT content alone. Although genomic signatures were originally based on dinucleotide frequencies [4], it has later been shown that tetranucleotide frequencies are better with respect to taxonomic classification [5,6]. In addition, the tetranucleotide based genomic signatures can distinguish between coding and non-coding regions within genomes which is difficult with dinucleotide based genomic signatures [6]. Since the genomic signature method varies little within genomes, it can also be used to detect special intra-genomic DNA regions [5,7-9]. Such regions may include highly conserved genes, such as rRNA operons, as well as horizontally transferred DNA such as pathogenicity islands [5,8,10,11].

Genomic signatures are presumed to be shaped by factors such as DNA structure, restriction and transcription systems, base-stacking energies, replication and repair, and more [12]. To what degree these factors influence the genomic signature, however, has not been resolved [13]. The aim of this study was therefore to explore the origin and the strength of the phylogenetic signal of genomic signatures. In addition, we analyzed how the genomic signature was affected by mutational pressure, measured as the oligonucleotide usage variance (OUV, equation (6) in the methods section) between genomic oligonucleotide frequencies and corresponding mononucleotide approximated oligonucleotides frequencies [14].

The OUV measure calculates the deviance between genomic oligonucleotide frequencies and approximated oligonucleotide frequencies using the considered oligonucleotide's mononucleotide frequencies. This reflects how genomic oligonucleotide usage is biased compared to what is expected from genomic AT content. In effect, since each considered oligonucleotide frequency is approximated by its corresponding mononucleotide frequencies, complete independence is assumed between the nucleotides in the approximated oligonucleotide. Hence, the OUV measure approximates genomic oligonucleotide frequencies using genomic AT content. Large OUV values are therefore indicative of strong bias or selective pressure, while low OUV values are associated with mutagenesis.

Additionally, we compared the phylogenetic signal of the genomic signature to factors such as AT content, growth temperature, habitat, and chromosome size. To do this, 867 prokaryotic chromosomes and plasmids larger than 100 kb were compared pair-wise. The method of choice was hexanucleotide frequency based genomic signatures, since that particular method has been found to reflect a stronger phylogenetic signal than both di- and tetranucleotide based genomic signatures [5]. Since the genomic signatures are metric-based, bootstrapping or related methods are not possible [13]. K-means hierarchical clustering was therefore performed on the resulting pair-wise comparisons of all included DNA sequences. A multinomial regression model was subsequently fitted to the different cluster groups to assess the individual influences exerted by the different factors mentioned above.

## Results

### Bias in oligonucleotide usage

OUV scores were calculated for observed di-, tetra- and hexanucleotide frequencies for all DNA sequences and fitted to regression models as response variables with genomic AT content as the predictor. The equations resulting from the regression models can be found in Table 1, where it can also be observed that significant association between AT content and OUV scores were found for all measures. Highest '% coefficient of determination', or *R*^2 ^score, was achieved for the hexanucleotide frequency based model, while the lowest score was found for the dinucleotide frequency based model.

**Table 1:** Regression models of genomic di-, tetra- and hexanucleotide frequencies and AT content

**DNA word size**	**Regression equations**	**Coefficient of determination**	**Significance**
Dinucleotides	Y_2 _= exp(-6.42-8.64X_AT _+ 6.59X^2^_AT_)	*R*^2^* = 0.17*	*p < 0.001*
Tetranucleotides	Y_4 _= exp(-8.85-14.73X_AT _+ 12.39X^2^_AT_)	*R*^2^* = 0.33*	*p < 0.001*
Hexanucleotides	Y_6 _= exp(-11.74-21.94X_AT _+ 19.40X^2^_AT_)	*R*^2^* = 0.46*	*p < 0.001*

### Pairwise comparisons of genomes using genomic signatures

**Figure 1:**
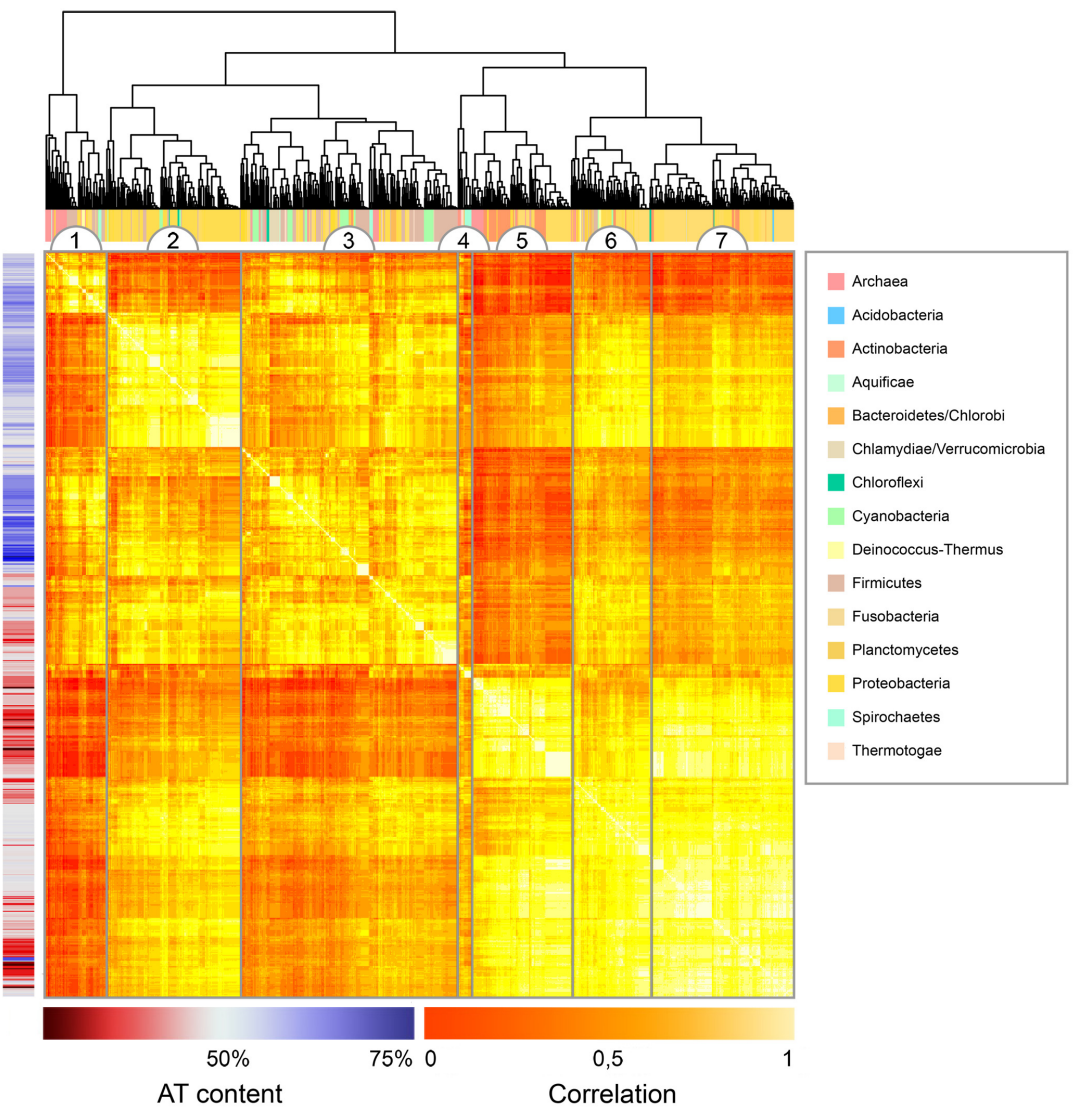
**Cluster diagram of 867 prokaryotic genomic DNA sequences compared pair-wise using hexanucleotide-based genomic signatures**. 867 prokaryotic genomic DNA sequences were compared pair-wise with hexanucleotide-based genomic signatures. Hierarchical clustering was performed on the resulting 867 × 867 correlation matrix using average linkage and Euclidean distance. The cluster diagram was grouped into different segments, Groups 1-7, based on the cluster-tree which reflected how the prokaryotic DNA sequences compared pair-wise. Lighter colors mean higher correlation scores, and thus closer similarity between the compared genomes. The multi-colored horizontal bar on top indicates each chromosome's respective phylum, while the vertical red and blue coloured bar shows AT/GC content, where red means GC content larger than 50% and blue AT content larger than 50%. Groups 5 and 7 are mainly populated with free-living, GC rich, prokaryotes with diverse metabolic capabilities. Groups 1 and 3 consist predominantly of AT rich and host-associated archaea and bacteria, while group 2 and 6 consisted mainly of larger host-associated *γ-Proteobacteria*. Group 4, was the smallest and most dissimilar group, consisting of many extremophiles.

**Figure 2:**
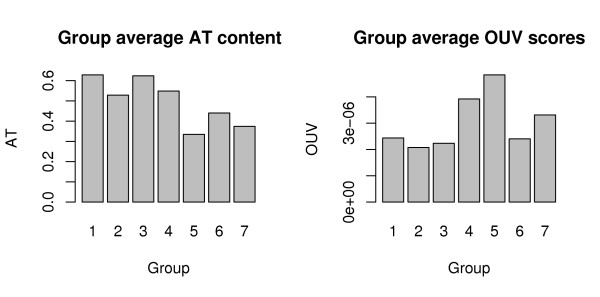
**Average AT scores and OUV content in cluster groups**. The graphs shows average AT content (left) and OUV scores (right) on the vertical axis, for each group on the horizontal axis. High OUV scores indicate strong bias in genomic hexanucleotide usage, while low scores imply more random DNA composition. Free-living archaea and bacteria (groups 5 and 7) obtain higher average OUV scores than host-associated (groups 1 and 3), indicating pronounced differences in mutational pressures in the respective environments. Average AT content was considerably higher in the host-associated groups than in the free-living.

The prokaryotic DNA sequences compared pair-wise using hexanucleotide-based genomic signatures were analyzed using cluster and multinomial regression analysis. Figure 1 shows the result of the cluster analysis and an overview of the different groupings. The full cluster diagram containing all the names of the included organisms can be found in additional file 1. A graph depicting average OUV scores and AT content for each group can be found in Figure 2.

The cluster diagram was divided into seven major groups, named groups 1 to 7, based on the cluster diagram in Figure 1. The most varied groups in terms of phyla were 1 and 3. Both had, on average, similar AT content and OUV scores. Many of the organisms in these groups were host-associated AT-rich bacteria like *Buchnera *spp., *Mycoplasma *spp., *Staphylococcus *spp., *Streptococcus *spp., the *Bacillus cereus *group [15], *Clostridium *spp. [16], *etc*.

Groups 2 and 6 contained larger host associated bacteria predominantly from the *γ-Proteobacteria *group. Average OUV scores were similar to groups 1 and 3, while AT content was lower (Figure 2).

Groups 5 and 7 contained metabolic diverse and free-living Proteobacteria and Actinobacteria. From Actinobacteria we found genera such as *Rubrobacter, Mycobacterium, Nocardia, Frankia, Rhodococcus, Thermobifida, Bifidobacterium, Streptomyces, Symbiobacterium, Propionibacterium, Leifsonia *and *Corynebacterium*. The Proteobacterial phylum was represented with *α- *and *β-Proteobacterial *genomes including genera such as *Caulobacter, Rhodobacter, Novosphingobium, Bradyrhizobium, Azoarcus, Burkholderia *and *Rhodopseudomonas*. The average AT content was lowest in these groups while the OUV score was highest.

Group 4 was the smallest of the groups discussed, and contained only twelve genomes. Both average AT content and OUV scores were fairly high compared to the other groups. The group obtained, on average, low correlation scores with the other groups and was therefore treated as a separate group. Members of the group included *Thermotoga *spp., *Rubrobacter × ylanophilus*, *Methanopyrus kandlerii*, *Methanosaete thermophila*, *Thermococcus kodakaraensis*, *Parabacteroidetes distasonis *and *Leptospira *spp.

### The model

The different cluster groups were fitted as a categorical variable to a regression model using the factors: genome size, AT content, OUV, phyla, growth temperature, oxygen requirement, and habitat. In Table 2, it can be concluded, based on the AIC (Akaike's Information Criterion) and McFadden *R*^2 ^statistics, that a fairly good model, with high explanatory power, was obtained for the cluster groups. AT content was the factor that had the largest impact on the model, followed by phyla. Although the phyla factor improved the model considerably, the effect was noticeably weaker than what was observed by including AT content as a factor. Genome size, habitat and growth temperature were also significant factors, but the regression model improved only slightly in terms of AIC and *R*^2 ^scores. The OUV and oxygen requirement factors were the weakest predictors. A factor specifying whether the organisms were pathogenic was originally included in the model, but was not found significant and was therefore removed.

**Table 2:** Polychotomous regression model with added predictors to the far left

**Model components**	**Log-Likelihood**	**McFadden R^2^**	**ΔAIC**	**AIC**
Model 0: constant	-1534	0	0	3080
Model 1: Size	-1475	0.04	95	2985
Model 2: AT content	-796	0.48	1333	1652
Model 3: OUV	-775	0.49	30	1622
Model 4: Phyla	-433	0.72	455	1167
Model 5: Oxygen req.	-414	0.73	15	1152
Model 6: Habitat	-360	0.77	61	1091
Model 7: Temperature	-320	0.79	56	1035
Final model	-320	0.79	-	1035

The table shows a forward fitting of a set of predictors to the response variable representing the cluster groups.

It should be noted that there is some co-linearity between the factors in the regression model. The predicted influences of each factor in Table 2 may therefore not be completely accurate. The model should rather be considered as a more general estimate of the influences exerted by the different factors included.

## Discussion

### Selection pressure as measured by OUV

The calculation of OUV gives an indication of how random or biased the occurrences of oligonucleotides are in genomes (See Methods section, as well as [14,17]). Since only AT content was used to approximate genomic oligonucleotide usage, the model assumes complete independence between all nucleotides in the genome. Thus, lower OUV scores imply more random DNA composition, and high variance scores can be taken to mean that stronger selection forces are affecting the distribution of genomic oligonucleotide frequencies. Table 1 show that the differently sized oligomers are differently affected by selective forces as measured by OUV. In other words, OUV, measured using longer oligonucleotides, are more strongly associated with genomic AT content than OUV based on shorter oligomers.

### Analysis of the model

The multinomial regression model gives a rough prediction of influences determining similarity with respect to the genomic signature discussed here. Figure 1 indicates that AT content and phyla appears to be associated with group formation in the cluster diagram.

It has been observed [5] that genomic signatures grouped organisms progressively better, with respect to 16S rRNA based phylogeny, when the oligonucleotide size increased. In addition, the number of wrong species identifications (false positives) dropped [5]. Hexanucleotide based genomic signatures were therefore the only measure considered in this study. Even though the genomic signature based comparisons cannot be directly compared to tree based phylogenetic methods [13], the over 150 phylogenetic groups found in the cluster diagram (see Figure 1) imply that the genomic signatures have limited taxonomic scope below genus level compared to rRNA based methods. rRNA based methods, on the other hand, are not optimal to compare strains [18]. Therefore, as has been stated previously [6,13,19], the genomic signature is a measure to be used together with rRNA-based methods.

The categorical factors included in the model must be considered as rough, giving only inferential knowledge. This is especially noticeable in the factor describing a genome's habitat, where many host-associated genomes may be found in multiple environments and vice versa.

Table 2 indicates the factors influencing comparisons based on genomic signatures, with AT content being the strongest. Habitat, oxygen requirement, and growth temperature were also significant factors, implying that signature differences may be found in strains and closely related species living in different environments or having dissimilar growth temperature and oxygen requirements. Oxygen requirement has been associated with AT content so this result was not unexpected [20]. Interestingly, growth temperature was found significant. This finding was of some interest due to the difficulty in establishing a link between base composition and growth temperature [21,22]. Our finding may indicate a sophisticated association between growth temperature and genomic base composition not easily detected with more traditional statistical methods.

A model was also created with the addition of a pathogenicity factor. This factor was included since it is assumed that pathogenic bacteria exchange DNA with the surroundings more often than non-pathogenic ones [23]. The pathogenicity factor was not found significant, and was therefore removed from the final model.

### Analysis of the cluster groups

Figure 1 show that Groups 5 and 7 consists of both Actinobacteria and Proteobacteria closely clustered together. Since the different phyla cluster more closely together than other Proteobacteria it may be deduced that forces are at work giving similar oligonucleotide preference for very distantly related bacteria. Although these bacteria are all GC rich, it should be noted that the genomic signatures are normalized by AT content (see Formula (2), Methods section) in the sense that genomic oligonucleotide frequencies are compared to AT content. Hence, genomic signatures give a measure of how oligonucleotide frequencies are over- and underrepresented in a genome compared to what is expected from AT content alone, which, in effect, should remove any bias from mononucleotide frequencies. The organisms in Groups 5 and 7 are predominantly free-living, mostly found in soil, with a diverse set of metabolic capabilities. Although the genomes in groups 5 and 7 are varied in terms of phyla, they share many of the other factors found in Table 2. For instance, the genomes in both groups 5 and 7 have comparable AT content, genome sizes, lifestyle and growth temperature. Despite these differences, DNA composition usually remains similar for closely related species and strains. This is also reflected by the genomic signature. At the genus level and below, however, DNA compositional differences become more pronounced. It has recently been proposed that bacteria only rarely change habitat and when they do it may have profound effects on DNA composition [24]. Our results, on the other hand, indicate that distantly related organisms may adopt similar DNA composition when they are subjected to comparable selective forces, as measured by the factors used in the regression model, at least in terms of the genomic signature.

By clustering bacteria according to codon usage it was found that genomes grouped according to their respective habitat and life-style [25]. Although the hexanucleotide based genomic signatures gave clear and distinct clusters of soil/free-living bacteria, other niche specific groups similar to the ones found using codon bias [25] were not detected. It should be stated that the methods employed here are not related to the codon bias-based methods described in [25]. Codon bias is strongly associated with AT content [25], while genomic signatures are normalized with respect to AT content. In other words, genomic signatures are not directly associated with AT content, in contrast to codon bias measures, but indirectly as the regression models show.

Figure 1 shows that the groups compare differently in terms of correlation scores in the sense that some groups are more similar than others. These observed similarities might illustrate an evolutionary transition from free-living (Groups 5 and 7) to host-associated life styles (Groups 2 and 6) ending up intracellular (Groups 1 and 3). The hypothesized direction from a free-living environment to a host-associated is based on average OUV scores from the different groups (Figure 2) where the free-living bacteria were seen to have, on average, more biased oligonucleotide usage than the host-associated. Groups 1 and 3 obtained the lowest variance scores of all groups, indicating a more 'random' genomic oligonucleotide distribution, and hence DNA composition, in the host-associated Proteobacteria compared to the free-living. The more random DNA composition is presumably due to increased mutation rates caused by the loss of DNA repair systems [26,27]. In addition to the host associated and Gram-negative Proteobacteria, the Gram-positive and pathogenic Actinobacterium *Tropheryma whipplei *(the causative agent of Whipple's disease) is also present in Group 3. This bacterium is presumed to have undergone genome reduction [28], indicating a possible niche-specific bias in oligonucleotide distributions.

The above examples illustrate that prokaryotic DNA composition, expressed using hexanucleotide-based genomic signatures, can be similar regardless of kinship. The similar DNA composition is, according to our results, a consequence of a collection of factors having acted on the genomes. Thus, genomic analyses of organisms undergoing evolutionary transition between different environments may give many important clues concerning how differences in DNA composition may arise in closely related organisms.

## Conclusion

Our results, based on hierarchical clustering and multinomial regression, indicate that genomes compared using genomic signatures are primarily grouped according to AT content. In the model presented, AT content was more strongly associated with the clustered groups than taxonomy. Taxonomy was, in turn, found to be more strongly linked to the clustered groups than the other significant factors. The remaining factors found to significantly affect the regression model were, in order of importance, genome size, habitat, temperature, selection bias (OUV) and oxygen requirement. It can therefore be concluded that the genomic signature in prokaryotes is influenced by many factors which may explain the limited phylogenetic scope below genus level.

## Methods

All genomic DNA sequences were obtained from the NCBI genome database [29] together with information about the different organisms. Additional information can also be found in additional file 2.

The computer programs used to generate the results were made according to the explanations given below. The following notation will be used throughout:

Let (*w*_1_*w*_2_..*w*_*n*_)_*i*_, represent an oligonucleotide (*n*-mer) with 1 ≤ *i *≤ *N *= 4^*n *^possible combinations. The function

(1)

gives the overlapping empirical frequency of the oligonucleotide (*w*_1_*w*_2_..*w*_*n*_)_*i*_, with respect to the DNA sequence *Z *= {*w*_1_*w*_2_..*w*_*s*_}, where *S *is much larger than *n*.

This means that:



The hexanucleotide-based relative abundances can then be calculated as follows:

(2)

Where 1 ≤ *i *≤ *N *= 4^*n*^

The genomic signature is then found by comparing two genomic DNA sequences with the Pearson correlation formula:

(3)

*N *= 4^*n *^designates the total number of possible DNA word combinations, with

(4)

And

(5)

The nucleotides *w*_*l*_, 1 ≤ *l *≤ 6, in the denominator of equations (4) and (5), are the corresponding nucleotides in the *i*^th ^hexanucleotide *w*_1_*w*_2_*w*_3_*w*_4_*w*_5_*w*_6_.

The following formulas



represent the average hexanucleotide relative abundance values.

Hierarchical clustering based on Euclidean distance was performed on the resulting symmetric 867 × 867 correlation matrix. Average linkage was used to put emphasis on the closest matches based on group similarities.

Oligonucleotide usage variance (OUV) can be considered as a measure of oligonucleotide frequency bias, or selection pressure on the genomic DNA composition, and was calculated according to the given formula for each chromosome:

(6)

The function *M*_0_*((w*_1_*w*_2_...*w*_*n*_*)*_*i*_*) *approximates oligonucleotide frequencies with the corresponding mononucleotide frequencies:

(7)

The formula implicitly assumes that each nucleotide in the approximated *n-*mer is independent of the neighbouring nucleotides. In addition, equation (7) assumes that genomic oligonucleotide frequencies are only influenced by AT content, which means that low values can be interpreted as random mutations carrying little or no information. High variance values, on the other hand, mean that substantial information is carried by the oligonucleotide being approximated.

Linear regression analysis was performed between OUV for di-, tetra-, and hexanucleotide frequencies (response variable) and genomic AT content (predictor variable) using log transformation. *R*^2 ^designates '% coefficient of determination'.

A conditional logistic multinomial (polychotomous) regression model was fitted to asses the individual influences of predictors: genome size, AT content, OUV, phyla, oxygen requirement, habitat, growth temperature and pathogenicity, with the cluster groups as the response variable. The AIC and McFadden *R*^2 ^statistics were used as indicators of the quality of the fitted model. The following multinomial logistic regression model was run in the statistical program R using the package *nnet*:



The response variable "Groups" is a categorical variable consisting of the different cluster groups (see Figure 1). The predictors Phyla, Oxygen, Habitat and Growth temperature were also categorical factors, while Size, AT and OUV were numerical factors. The Oxygen factor consisted of the categories: aerobic, anaerobic and facultative. Habitat consisted of the categories: host-associated, multiple, specialized, terrestrial, and aquatic, while the growth temperature factor consisted of the following categories: psychrophilic, mesophilic and thermophilic. This information was taken from the NCBI website . The regression model converged after 220 iterations. Assessment of statistical significance was carried out with the *car *package.

All regression models were statistically significant with the significance level set to *p < 0.001*.

## Authors' contributions

JB planned the project, wrote the computer programs and the manuscript. ES contributed to the statistical analyses and critically revised the manuscript. DU drafted and critically revised the manuscript and analyzed the data. All authors read and approved the final manuscript.

## Supplementary Material

Additional file 1 **Genomic signature based cluster diagram**. JPG file containing 867 labelled prokaryotic DNA sequences compared pair-wise using hexanucleotide-based genomic signatures, and clustered using hierarchical clustering. Click here for file

Additional file 2 **Data file**. Excel file containing all 867 prokaryotic chromosomes and plasmids larger than 100 kb along with the corresponding list of genomic properties and phyla. Click here for file
